# Temperature- and wavelength-insensitive parametric amplification enabled by noncollinear achromatic phase-matching

**DOI:** 10.1038/srep36059

**Published:** 2016-10-27

**Authors:** Daolong Tang, Jingui Ma, Jing Wang, Bingjie Zhou, Guoqiang Xie, Peng Yuan, Heyuan Zhu, Liejia Qian

**Affiliations:** 1 Key Laboratory for Laser Plasma (Ministry of Education), Department of Physics and Astronomy, Collaborative Innovation Center of IFSA (CICIFSA), Shanghai Jiao Tong University, Shanghai 200240, China; 2 Shanghai Engineering Research Center of Ultra-precision Optical Manufacturing, Department of Optical Science and Engineering, Fudan University, Shanghai 200433, China

## Abstract

Optical parametric chirped-pulse amplification (OPCPA) has been demonstrated to be a promising approach for pushing femtosecond pulses towards ultra-high peak powers. However, the future success of OPCPA strongly relies on the ability to manipulate its phase-matching (PM) configuration. When a high average power pump laser is involved, the thermal effects in nonlinear crystals induce phase-mismatch distortions that pose an inherent limitation on the conversion efficiency. Here, we demonstrate that the noncollinear configuration previously adopted for wavelength-insensitive PM can be employed for temperature-insensitive PM when the noncollinear angle is properly reset. Simultaneous temperature- and wavelength-insensitive PM is realized for the first time by imposing such a temperature-insensitive noncollinear configuration with an angularly dispersed seed signal. Based on the lithium triborate crystal, the proposed noncollinear achromatic PM has a thermal acceptance 6 times larger than that of the conventional wavelength-insensitive noncollinear PM and has a sufficient spectral acceptance to support pulse durations of ~20 fs at 800 nm. These achievements open new possibilities for generating ultra-high peak power lasers with high average power.

Ultrafast lasers with high peak and average powers are reliable tools for micromachining^1^, strong-field physics^2^ and applications in biology, medicine and materials sciences^3^,^4^,^5^. Recently, the peak power has been boosted to hundreds of terawatts or even petawatt levels by means of Ti:sapphire chirped-pulse amplification^6^ (CPA) or optical parametric chirped-pulse amplification^7^,^8^ (OPCPA). The average power, however, remains restricted to several tens of watts^9^,^10^, which is much lower than the desirable level (e.g., nearly kilowatts for efficient high-order harmonic generation^11^) for revolutionizing ultrafast science. For Ti:sapphire CPA systems, the major problem affecting average power scaling is the unavoidable thermal lens effect resulting from energy storage in the gain media. Alternatively, OPCPA has great potential to improve the average power because of the instantaneous nature of the nonlinear process and may represent a promising candidate for obtaining ultrafast lasers with high peak and average powers^12^.

The phase-matching (PM) condition, i.e., the momentum conservation in nonlinear interactions, is the intrinsic factor governing the energy-conversion efficiency and bandwidth in OPCPA. However, the PM is sensitive to both the wavelength and temperature in collinear PM, resulting in limited spectral and thermal acceptance. As a common practice, the noncollinear configuration provides the possibility of wavelength-insensitive PM, supporting the amplification of ultrashort pulses of ~20 fs or less^13^,^14^,^15^,^16^,^17^,^18^,^19^. Besides, another promising approach for generating high-power few-cycle pulses is parametric amplification in the frequency domain^20^. In contrast, temperature-insensitive PM has never been achieved in OPCPA. Thermal effects in nonlinear crystals remain the major restriction limiting high average power OPCPA. Although OPCPA does not involve energy storage, absorption caused by material impurities is inevitable, particularly in the ultraviolet and mid-infrared spectral regions. For example, in the widely used beta-barium borate crystal, the absorption coefficients are ~0.01 cm^−1^ at 0.532 μm and ~0.5 cm^−1^ at 2.55 μm^21^. The absorption of the interacting waves non-uniformly heats the crystal and consequently induces phase-mismatch among the spectra, which is more severe in the high average power regime^22^. This type of phase-mismatch distortions degrades the conversion efficiency and distorts the spectral and temporal profiles of the signal pulses and thus places an inherent limitation on power scaling in OPCPA.

Aiming for the generation of high peak and average power ultrafast lasers, here we propose an OPCPA PM configuration that is insensitive to temperature and wavelength simultaneously. Figure 1 compares three PM configurations for OPCPA. In collinear PM (Fig. 1(a)), in which the pump and signal are collinearly injected into the crystal, the PM is sensitive to both the temperature and wavelength, as indicated by the wavelength tuning with temperature variation in the gain profile. Noncollinear PM (Fig. 1(b)) involves separating the pump and signal with a proper angle for wavelength-insensitive PM. In this system, the gain is almost independent of the wavelength over a broader range compared with collinear PM. However, because of the lack of control parameters for achieving temperature-insensitive PM, a rapid drop in the gain profile is inevitable once the temperature deviates from the operating temperature. As reported here, the noncollinear configuration can be transformed from conventional wavelength-insensitive PM to temperature-insensitive PM, which is governed by the noncollinear angle. Based on this process, we propose a noncollinear achromatic PM (NAPM) configuration that combines the temperature-insensitive noncollinear configuration and the achromatic PM using an angularly dispersed seed signal (Fig. 1(c)). Consequently, the proposed NAPM can be insensitive to both temperature and wavelength and may be used to boost ultrafast lasers towards unprecedented high average powers.

## Results

### Characteristics of NAPM

As for all parametric processes, efficient OPCPA conversion requires that the PM condition be satisfied, i.e., the phase-mismatch (Δ*k*) among the interacting waves must be eliminated. In collinear PM, the phase-mismatch can be expressed as


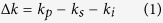


where *k*_*p*,*s*,*i*_ are the wave-vectors of the pump, signal and idler, respectively. Whereas the PM condition can typically be satisfied by angle tuning in nonlinear crystals, an additional control parameter is essential for achieving either wavelength-insensitive PM or temperature-insensitive PM. Traditionally, the noncollinear configuration is adopted to achieve wavelength-insensitive PM, which leads the phase-mismatch as


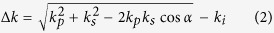


where *α* is the noncollinear angle between the pump and signal. The wavelength-insensitive PM requires vanishing the first derivative of the phase-mismatch with respect to the signal frequency:


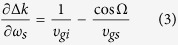


where *υ*_*gs*_ and *υ*_*gi*_ are the group-velocities, and Ω is the angle between the idler and signal. Wavelength-insensitive PM can be achieved when the group-velocities of the signal and idler are equal to each other along the signal propagation direction^23^:


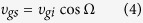


Notably, the role of noncollinear configuration is not fundamentally specific to wavelength-insensitive PM. Because of the strong relation between the PM condition and the noncollinear angle, the noncollinear angle is a degree of freedom that allows the manipulation of the noncollinear configuration. Specifically, we find that the noncollinear configuration can be set to achieve temperature-insensitive PM:


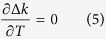


At an appropriate noncollinear angle, the above requirement can be satisfied. However, the noncollinear angle for temperature-insensitive PM generally differs from that required for wavelength-insensitive PM, and another compensating parameter is needed to support simultaneous wavelength-insensitive PM. Angular dispersion of the seed signal may be suitable for this purpose. It requires


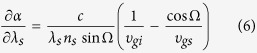


where *λ*_*s*_ and *n*_*s*_ are the wavelength and refractive index of the signal, respectively. *c* is the velocity of light in vacuum. The right-hand term in Eq. (6) involves the group-velocity mismatch caused by the deviation from the angle for wavelength-insensitive PM, which can be compensated by an adequate angular dispersion of the seed signal, as shown in the left-hand term. Previously, the seed signal with angular dispersion has been proposed for ultra-broadband parametric amplification^24^ and pulse durations of 5.8 fs has been experimentally demonstrated^25^. The needed angular dispersion can be introduced with a grating^24^,^25^ or grating pair^26^, etc. The exact amount of angular dispersion can be measured with the aid of spectrally resolved interferometry^27^ and the re-collimation of the amplified signal can be achieved by angular-dispersion-compensation scheme^28^. Because the proposed PM configuration is a combination of the temperature-insensitive noncollinear configuration and the achromatic PM using an angularly dispersed seed signal, we term it NAPM.

To illustrate the characteristics of the NAPM by numerical simulations, we choose the following OPCPA parameters: femtosecond Ti:sapphire pulses at 800 nm and picosecond pulses at 532 nm serve as the signal and pump pulses, respectively. Lithium triborate (LBO)^29^ is selected as the nonlinear crystal because of its favourable features of large aperture, high damage threshold and small spatial walk-off. In this article, type-I PM in the xy plane is assumed, in which both the signal and the idler are ordinary polarized, and the pump is extraordinary polarized.

**Figure 2:**
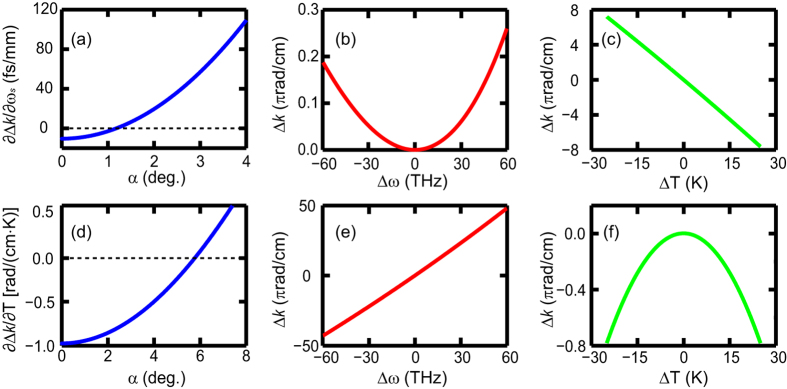
The roles of the noncollinear angle in manipulating the OPCPA PM. (**a**) The first derivative of the phase-mismatch with respect to signal frequency (∂Δ*k*/∂*ω*_*s*_) versus the noncollinear angle *α*. The dependence of the phase-mismatch (Δ*k*) on the signal frequency (**b**) and temperature (**c**) when the noncollinear angle is tuned to 1.18° to set ∂Δ*k*/∂*ω*_*s*_ = 0. (**d**) The effect of the noncollinear angle in manipulating the first derivative of the phase-mismatch with respect to temperature (∂Δ*k*/∂*T*). Δ*k* versus the signal frequency (**e**) and temperature (**f**) when the noncollinear angle is reset to 5.77° to eliminate ∂Δ*k*/∂*T*. The operating temperature was fixed at 323 K.

Figure 2 shows the roles of the noncollinear angle in manipulating the OPCPA PM. We first consider wavelength-insensitive PM in the noncollinear configuration. In Fig. 2(a), we plot the first derivative of the phase-mismatch with respect to the signal frequency (∂Δ*k*/∂*ω*_*s*_) versus the noncollinear angle *α*. When the group-velocities of the signal and idler are matched (*α*  = 1.18°), ∂Δ*k*/∂*ω*_*s*_ vanishes exactly, and wavelength-insensitive PM is achieved, corresponding to the conventional noncollinear PM^30^. As a result, the dependence of the phase-mismatch Δ*k* on the frequency, which is dominated by the second derivative of the phase-mismatch term (i.e., ∂^2^Δ*k*/∂*ω*_*s*_^2^), is very weak (|Δ*k*| < 2π rad/cm), as shown in Fig. 2(b). However, the phase-mismatch is linearly dependent on temperature with a large slope (|∂Δ*k*/∂*T*|·|Δ*T*| > 2π rad/cm), indicating that the PM is highly sensitive to temperature, as shown in Fig. 2(c). To verify the feasibility of the noncollinear configuration to achieve temperature-insensitive PM, in Fig. 2(d), we plot the first derivative of the phase-mismatch with respect to temperature (∂Δ*k*/∂*T*) versus the noncollinear angle *α*. When the noncollinear angle is properly reset (*α*  = 5.77°), ∂Δ*k*/∂*T* vanishes exactly, and temperature-insensitive PM is achieved. Thus, the dependence of the phase-mismatch on temperature, which is dominated by the second derivative of the phase-mismatch term (i.e., ∂^2^Δ*k*/∂*T*^2^), is very weak, as shown in Fig. 2(f). Figure 2(e) shows the dependence of phase-mismatch on the frequency. Because the noncollinear angle in this case largely differs from that required for wavelength-insensitive PM, group-velocity mismatch between the signal and idler arises. Consequently, the phase-mismatch is linearly dependent on the frequency, and the PM is highly sensitive (|Δ*k*| > 2π rad/cm) to the wavelength.

**Figure 3:**
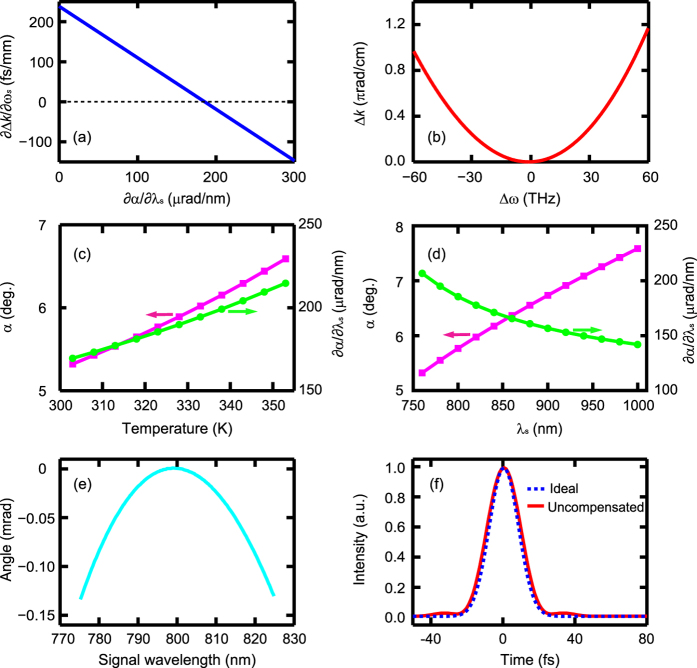
The required noncollinear angle and angular dispersion for the NAPM. (**a**) The influence of the angular dispersion (∂*α*/∂*λ*_*s*_) on ∂Δ*k*/∂*ω*_*s*_ when ∂Δ*k*/∂*T* = 0. (**b**) The dependence of the phase-mismatch on the signal frequency with the internal angular dispersion of 186 μrad/nm. The operating temperature was fixed at 323 K. The feasibility of the NAPM at different temperatures (**c**) and wavelengths (**d**). (**e**) The angle difference between the noncollinear angle for an accurate PM and its linear approximation in the NAPM. (**f**) Pulse characteristics after a propagation distance of 0.5 m. The “Ideal” case excludes the effect of the nonlinear angular dispersion, while the “Uncompensated” case denotes the case that the nonlinear angular dispersion is included and is uncompensated after propagation.

To ensure that the proposed NAPM is insensitive to the wavelength additionally, angular dispersion is further imposed on the seed signal. Figure 3(a) shows the dependence of ∂Δ*k*/∂*ω*_*s*_ on the angular dispersion at the noncollinear angle of 5.77°. ∂Δ*k*/∂*ω*_*s*_ is linearly dependent on angular dispersion. With an angular dispersion of ~186 μrad/nm inside the crystal, ∂Δ*k*/∂*ω*_*s*_ vanishes exactly, and wavelength-insensitive PM is achieved. This angular dispersion is easily obtained with a grating (with a groove density of ~300 line/mm) working in the Littrow configuration. Due to both the noncollinear configuration and angular dispersion of the seed signal, the newly generated idler is also angularly dispersed with a value of ~101 μrad/nm inside the crystal. Similar to the conventional wavelength-insensitive noncollinear PM, the phase-mismatch is also very weak and is mainly determined by ∂^2^Δ*k*/∂*ω*_*s*_^2^, as shown in Fig. 3(b). The NAPM can be constituted at different operating temperatures if the noncollinear angle and angular dispersion are properly set. Figure 3(c) shows the required noncollinear angle and angular dispersion for NAPM at different operating temperatures. Similar conclusions can also be drawn for the case with varied signal wavelengths, as shown in Fig. 3(d).

In parametric processes, the nonlinear angular dispersion may be introduced. It is then essential to address the effect of the nonlinear (i.e., high-order) angular dispersion, particularly in the case of large bandwidth. Since the signal amplification in OPCPA is largely governed by the PM condition, the nonlinear angular dispersion can be simply predicted by the PM property. To evaluate the amount of the nonlinear angular dispersion in the NAPM, Fig. 3(e) shows the angle difference between the wavelength-dependent noncollinear angle for an accurate PM and its first-order approximation (i.e., the imposed linear angular dispersion of ~186 μrad/nm on the incident signal). The nonlinear angular dispersion is dominated by a second-order angular dispersion of ~−0.42 μrad/nm^2^. To study its effect, we impose such amount of nonlinear angular dispersion on the signal pulse with a bandwidth of 50 nm (full width at half maximum) and consider its propagation evolution in free space. As we know, a linear angular dispersion induces a second-order group-velocity dispersion^31^, and the second-order angular dispersion will impose a fourth-order dispersion (i.e., a quartic phase on the spectrum) accordingly. If necessary, such a fourth-order dispersion can be well compensated by e.g. acousto-optic programmable dispersive filter. In practical systems, however, the effect of the nonlinear angular dispersion can be ignored. Figure 3(f) presents both the pulses with and without the nonlinear angular dispersion. Considering a propagation distance of 0.5 m (the typical size of a compressor and focusing system), the effect of the nonlinear angular dispersion will only stretch the pulse duration from an ideal value of 19 fs (transform-limited) to 20 fs. Furthermore, it is noteworthy that in the conventional wavelength-insensitive PM, such kind of noncollinear angular dispersion will also be induced with a comparable amount to that in the NAPM. In this wavelength-insensitive PM, few-cycle pulses have been widely demonstrated without the need of compensating the nonlinear angular dispersion^14^,^15^,^16^, which provides a further confidence for the feasibility of the proposed NAPM.

### OPCPA performance with the NAPM

To address the advantages of the NAPM over the wavelength-insensitive noncollinear PM in parametric amplification, both the conversion efficiency and the signal pulse profiles are numerically investigated based on the nonlinear coupled-wave equations^32^ (see Methods for detail). In the simulations, both the pump and chirped signal durations (full width at half maximum) are designated to be 10 ps with a Gaussian profile. The pump intensities are selected to ensure the same nonlinear length^33^ *L*_*NL*_ = *n*_*p*_*λ*_*p*_/(2*πd*_*eff*_ *A*_*p*0_), where *n*_*p*_ is the refractive index, *λ*_*p*_ is the pump wavelength, *d*_*eff*_ is the effective nonlinear coefficient and *A*_*p*0_ is the input pump field. The small signal gain is designed to be ~1000 with a crystal length of 15 mm. The intensity ratio (I_p_/I_s_) is fixed at 100:1 in both cases, which is typical for the high energy/final stage amplifiers.

**Figure 4:**
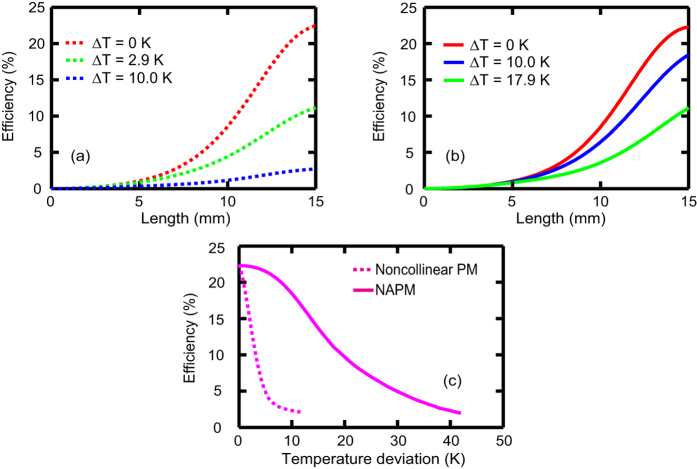
Signal efficiency evolution versus crystal length and temperature. Evolution of the efficiency inside the crystal at different temperatures in (**a**) the wavelength-insensitive noncollinear PM and (**b**) the NAPM. (**c**) The output efficiency versus temperature for the wavelength-insensitive noncollinear PM and NAPM. The crystal length (L) was fixed at 15 mm. The nonlinear length was 3 mm (0.2 L) in both cases.

Figure 4(a) presents the signal efficiency evolution in a 15-mm LBO crystal for the wavelength-insensitive noncollinear PM. At the operating temperature (ΔT = 0 K), the efficiency increases with increasing crystal length and reaches a maximum value of 22.4% at the chosen length of 15 mm. When the temperature deviates from the operating temperature (ΔT ≠ 0 K), signal amplification still occurs, but the efficiency drops significantly because of the thermal-induced phase-mismatch. The efficiency is degraded to a greater extent by larger temperature deviations. For instance, the output efficiencies decrease to only 11.2% and 2.3% at temperature deviations of 2.9 and 10.0 K, respectively. Similar behaviours can also be observed in the NAPM, as shown in Fig. 4(b). However, the thermal effects are markedly alleviated in the NAPM. The output efficiency remains 18.5% (11.2%) at a larger temperature deviation of 10.0 K (17.9 K), indicating desirable temperature-insensitive PM. Figure 4(c) shows the dependence of the output efficiency on temperature in the two PM schemes. The efficiencies decrease as the temperature deviation increases in both PM schemes; nevertheless, the efficiency decreases much more slowly in the NAPM. Quantitatively, the thermal acceptance is as large as 17.9 K in the NAPM, which is more than 6 times larger than that observed in wavelength-insensitive noncollinear PM (2.9 K). (The thermal acceptance is defined as the maximum temperature deviation when the output efficiency is no less than half of that at the operating temperature).

**Figure 5:**
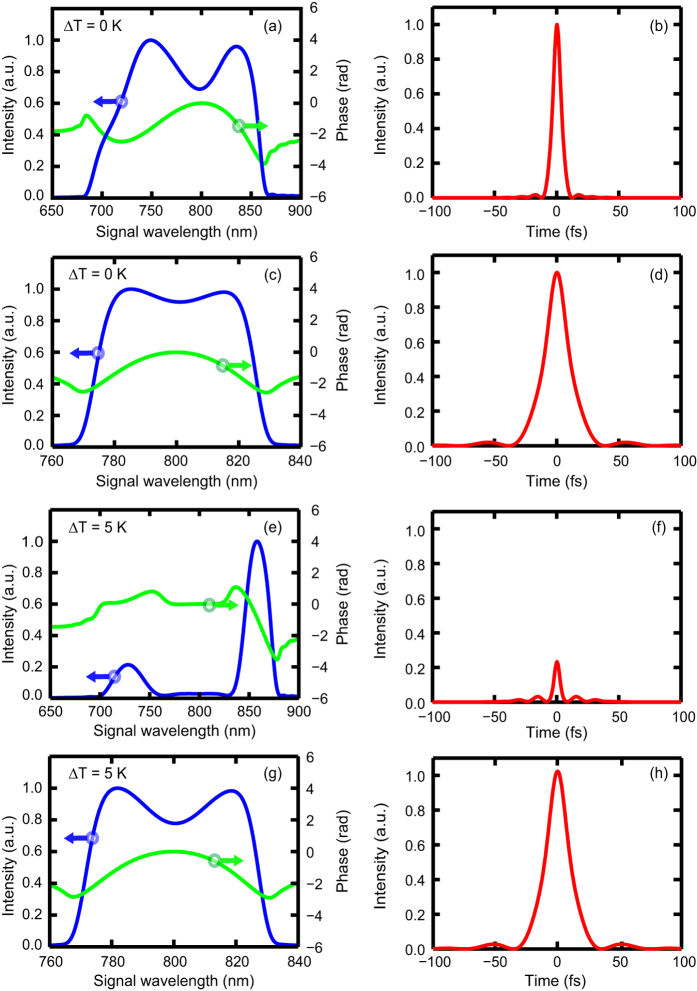
Spectral and temporal profiles of the amplified signal in the wavelength-insensitive noncollinear PM and NAPM. (**a**) The signal spectrum intensity and OPP in wavelength-insensitive noncollinear PM at ΔΤ = 0 K. (**b**) Transform-limited pulse in wavelength-insensitive noncollinear PM corresponding to the spectrum in (**a**). (**c**) The amplified signal spectrum in the NAPM at ΔΤ = 0 K. (**d**) Transform-limited pulse in the NAPM corresponding to the spectrum in (**c**). The amplified signal spectra and transform-limited pulses in the wavelength-insensitive noncollinear PM (**e**,**f**) and NAPM (**g**,**h**) at ΔΤ = 5 K. In (**b**,**d**,**f**,**h**), the intensity is normalized to the maximum intensity of ΔΤ = 0 K.

In addition to a larger thermal acceptance, the NAPM is also able to support the amplification of femtosecond pulses of ~20 fs because of the elimination of the ∂Δ*k*/∂*ω*_*s*_ term. For comparison, Fig. 5(a) shows the spectrum and optical parametric phase (OPP) of the amplified signal pulses in the wavelength-insensitive noncollinear PM at the operating temperature. If the OPP is compensated, the transform-limited pulse can be obtained with a duration of 7.7 fs, as shown in Fig. 5(b). Figure 5(c,d) show the amplified signal spectrum and pulse profile in the NAPM at the operating temperature. Because of the residual uncompensated ∂^2^Δ*k*/∂*ω*_*s*_^2^, the duration of the transform-limited pulse is ~20 fs in the NAPM. However, the advantages of the NAPM are revealed when the temperature deviates from the operating temperature, which is inevitable in high average power OPCPA. In this case, both the spectrum and pulse profile are influenced by the thermal-induced phase-mismatch. For instance, with a temperature deviation of 5 K, the spectrum in wavelength-insensitive noncollinear PM is severely distorted, as shown in Fig. 5(e). Because of the severe phase-mismatch, a large dip appears around 800 nm, indicating insufficient amplification. In the time domain, because of the low efficiency, the intensity decreases to 30% of that at the operating temperature, as shown in Fig. 5(f). In contrast, the NAPM is robust with respect to temperature. As seen from Fig. 5(g), the spectral distortion is almost negligible compared with that in the wavelength-insensitive noncollinear PM. Meanwhile, the signal intensity remains almost unchanged, as shown in Fig. 5(h), which is desirable for power scaling. Because of the short pulse duration and large thermal acceptance, ultrafast pulses with both high peak and average powers can be anticipated in the NAPM.

A proof-of-principle experiment was implemented for demonstrating the NAPM. We used the same pump and signal lasers systems as described in our previous work^34^,^35^. For the NAPM, the angular dispersion was controlled by tuning the parallel misalignment of the grating pair (1480 line/mm) in the stretcher. An image-relay telescope was adopted to maintain the angular dispersion, thus no spatial chirp was introduced and the temporal chirp was not altered. Both the NAPM and wavelength-insensitive PM configurations were characterized using 25-mm-thick LBO crystals. In the experiments, a temperature-controlled crystal oven was employed, which can heat up the crystal uniformly to a desired temperature over a temperature range of 313 to 453 K (accuracy of ± 0.1 K).

**Figure 6:**
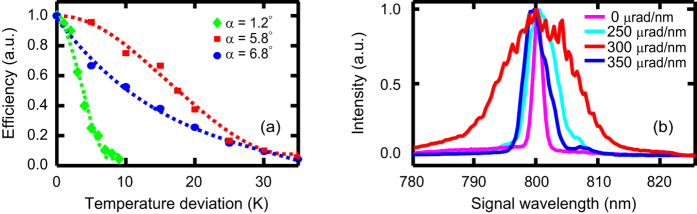
The effects of noncollinear angle and angular dispersion on the NAPM. (**a**) Measured signal efficiency versus temperature deviation for several noncollinear angles. Both the cases with noncollinear angle *α* = 5.8° and 6.8° correspond to the NAPM (crystal orientation: *θ* = 90°, *φ* = 51.2°), while the case with *α* = 1.2° corresponds to the conventional wavelength-insensitive PM (crystal orientation: *θ* = 90°, *φ* = 12.0°). In order to keep a similar gain and efficiency, the pump intensities in both the PM configurations were adjusted to ~4 GW/cm^2^ and ~2 GW/cm^2^, respectively. Lines are fitted for eyes. (**b**) Measured spectra of the amplified signal with different amount of external angular dispersion. The noncollinear angle was optimally fixed at 5.8°.

To illustrate the role of the noncollinear configuration in increasing the thermal acceptance, we studied the signal efficiency versus temperature for several noncollinear angles (Fig. 6(a)). At the operating temperature, signal efficiency of ~11% (~12%) was obtained in the NAPM (wavelength-insensitive noncollinear PM). Due to the large value of |∂Δ*k*/∂*T*|, the thermal acceptance in the wavelength-insensitive PM (*α* = 1.2°) was as small as ~3 K, which is in good agreement with the numerical results. As a result of the decreasing |∂Δ*k*/∂*T*|, the thermal acceptance became larger as the noncollinear angle was increased. In particular, maximum thermal acceptance (~17 K) was observed when |∂Δ*k*/∂*T*| was eliminated in the NAPM (*α* = 5.8°). As the noncollinear angle was further increased, the thermal acceptance became smaller owing to the non-vanishing |∂Δ*k*/∂*T*|. The effect of the angular dispersion on spectral bandwidth is shown in Fig. 6(b). Maximum spectral bandwidth was achieved when ∂Δ*k*/∂*ω*_*s*_ was eliminated at the optimum amount of angular dispersion (~300 μrad/nm outsider the crystal), while bandwidth narrowing occurred when the angular dispersion was not set properly.

## Discussion

In this article, we demonstrate a PM configuration for OPCPA that is insensitive to temperature and wavelength simultaneously, which is achieved by adopting a temperature-insensitive noncollinear configuration and applying angular dispersion to the seed. Numerical simulations and a proof-of-principle experiment based on LBO crystal prove that the thermal acceptance in the NAPM is more than 6 times larger than that in the conventional wavelength-insensitive noncollinear PM. Meanwhile, the NAPM is also able to support ~20 fs pulses and shows desirable thermal robustness. In addition, shorter pulses can be anticipated if the bandwidth of the seed signal is increased. The NAPM is not restricted to a specific wavelength and/or nonlinear crystal. Indeed, the NAPM can be achieved at different wavelengths if the noncollinear angle and angular dispersion are properly set. Yttrium calcium oxyborate^36^, another widely used crystal, is also a good candidate for NAPM in the near-infrared region in its xy plane. Furthermore, for other nonlinear conversions, such as sum frequency generation, such a temperature-insensitive PM is also valid. The demonstrated PM configuration may serve as a robust means for power scaling of various nonlinear interactions in wide spectral regions.

## Methods

### Nonlinear coupled-wave equations

Under the approximation of slowly varying envelope, the coupled-wave equations that govern the three-wave interaction are given by:


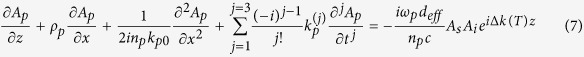



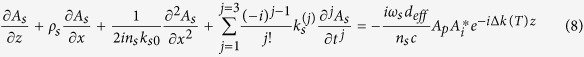



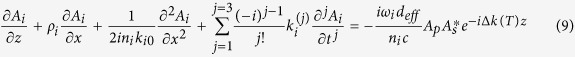



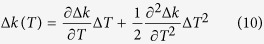


where *A*_*p*,*s*,*i*_ and *ω*_*p*,*s*,*i*_ are the envelopes and angular frequencies of the pump, signal and idler, respectively. *ρ*_*p*,*s*,*i*_ are the walk-off angles and *n*_*p*,*s*,*i*_ are the refractive indices. *k*_*p*0,*s*0*,i*0_ denote the wave-vectors in vacuum. *k*^(*j*)^ is the *j*th-order dispersion coefficient. It is noteworthy that the walk-off angles, the refractive indices, the wave-vectors and the dispersion coefficients are calculated at the central wavelengths and operating temperature as a common practice. *z* is the propagation direction. In the above equations, to include the effects of the noncollinear geometry, the transverse co-ordinate (*x*) that involves noncollinear geometry is taken into account, while diffraction in the other transverse co-ordinate (*y*) is neglected. This is reasonable because the beam diffraction-length is typically much longer than the crystal length. The dispersion effects on the three-wave interactions are given by the dispersion terms on the left side. Specifically, based on the crystal data provided by A. V. Smith *et al.*^37^, the first three terms of dispersion ∂Δ*k*/∂*ω*_*s*_, ∂^2^Δ*k*/∂*ω*_*s*_^2^, ∂^3^Δ*k*/∂*ω*_*s*_^3^ are 0 (0) fs/mm, 202 (44) fs^2^/mm, 858 (140) fs^3^/mm for NAPM (wavelength-insensitive PM) in LBO crystal, respectively. To simply illustrate the role of the thermal-induced phase-mismatch in amplification, we do not address the real temperature distribution inside the crystal. Alternatively, as shown in Eq. (10), thermal effects are considered by the temperature-dependent phase-mismatch Δ*k(T*) in the final exponential term. The temperature coefficients^37^ ∂Δ*k*/∂*T*, ∂^2^Δ*k*/∂*T*^2^ in the Taylor expansion are 0 (−0.93) rad/(cm·K), −7.8 × 10^−3^ (−2.2 × 10^−3^) rad/(cm·K^2^) for NAPM (wavelength-insensitive PM), respectively. To solve the coupled-wave equation numerically, the linear terms (dispersion, noncollinear geometry and diffraction) are calculated with symmetric split-step Fast Fourier Transform method, while the nonlinear interaction term is dealt with fourth-order Runge-Kutta method.

## Additional Information

**Publisher's note**: Springer Nature remains neutral with regard to jurisdictional claims in published maps and institutional affiliations.

**How to cite this article**: Tang, D. *et al.* Temperature- and wavelength-insensitive parametric amplification enabled by noncollinear achromatic phase-matching. *Sci. Rep.* **6**, 36059; doi: 10.1038/srep36059 (2016).

## Figures and Tables

**Figure 1:**
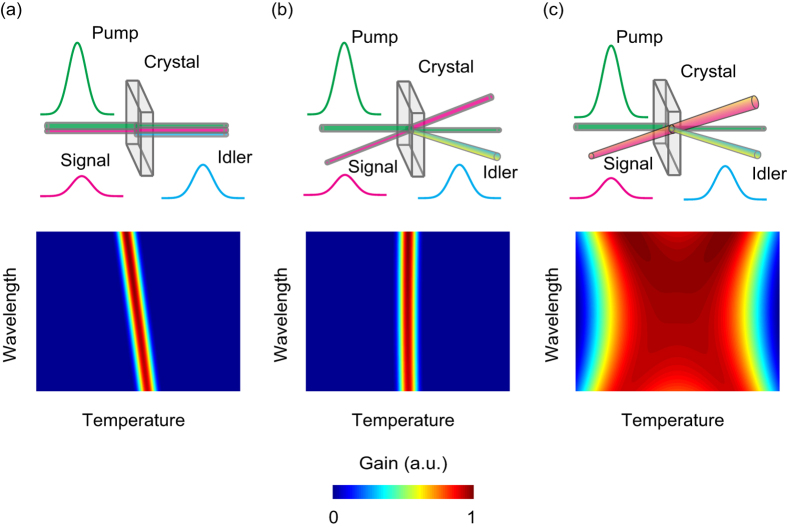
PM configurations for OPCPA. (**a**) Collinear PM. The pump and signal are collinearly injected into the nonlinear crystal. (**b**) Wavelength-insensitive noncollinear PM. The pump and signal are separated by a noncollinear angle. (**c**) NAPM. The pump and signal are separated by a noncollinear angle set for temperature-insensitive PM. Besides, the seed signal is angularly dispersed for achromatic PM. The second row shows the normalized gain profile versus temperature and wavelength corresponding to the three PM configurations in the first row.
